# The Hospital World

**Published:** 1910-11-05

**Authors:** 


### The Hospital World.

His Serene Highness Prince Alexander of Teck has accepted the Chairmanship of the Middlesex Hospital. The Duke of Teck will become a Vice-President of the hospital. His Serene Highness has received the following letters from their Majesties the King and Queen :—

Marlborough House, October 29, 1910.

Sir,—I have it in command to enclose herewith a cheque for one hundred guineas.

The King is graciously pleased to express a desire that this money shall be taken as a donation from his Majesty to the Middlesex Hospital in memory of the zeal, the skill, and the energy which the King's late brother-in-law, Prince Francis of Teck, showed on behalf of the funds of the hospital, and of which the Prince was mercifully spared to see the splendid results.

I have the honour to be, Sir, your Serene Highness's obedient servant,

W. Carington,
Keeper of his Majesty's Privy Purse.

His Serene Highness Prince Alexander of Teck, G.C.V.O., Chairman, the Middlesex Hospital.

---

Marlborough House, October 29, 1910.

Sir,—I have received the Queen's commands to forward to you a cheque for £100, which her Majesty wishes to contribute to the funds of the Middlesex Hospital in remembrance of the energy and keen interest displayed by her late brother, his Serene Highness Prince Francis of Teck, in that institution, and to express her Majesty's best wishes for the continued success of the hospital.

I have the honour to be, Sir, your Serene Highness's obedient servant,

E. W. Wallington,
Private Secretary to her Majesty the Queen.

Prince Alexander has not let the grass grow under his feet, for on the day his acceptance of the chairmanship was made known, a letter from him appeared in the *Times*, from which we take the following :—

Sir,—The last letter written by my late brother in connection with his work at the Middlesex Hospital was one in which he expressed his grateful thanks to those who had responded to his appeal for £20,000 with the object of removing the debt upon that institution.

He concluded his letter as follows :—

"But my task is not yet finished," and "until a steady and permanent addition of £7,000 per annum is made to the hospital's income, its financial position is not secure, and every third year the Governors will find themselves face to face with a crisis similar to that which has now happily been averted.

"It is my ambition to substitute, for such a hand-to-mouth administration as this, one which will provide the Governors with an income sufficient to meet the normal expenses of the year, and, directly I am able to do so, it is my intention to devote my time and energy to building up an adequate annual subscription and donation list."

This was his ambition, the purpose to which he intended to devote his life, and, had he lived, I feel sure that his efforts would have been crowned with success.

There is a general feeling . . . that no more appropriate tribute could be paid to his memory than by the establishment of an endowment fund which would produce £7,000 a year, the amount by which the normal annual expenditure exceeds the annual income.

I am conscious that the example which my brother set . . . is difficult to follow, but I am determined . . . to carry on the work which was so dear to him.

I have accordingly accepted the invitation of the

Governors to become their Chairman; I take up my brother's work where he left off, and it is my earnest hope that I may ultimately achieve the object he sought to accomplish.—Yours truly,

Alexander George of Teck.

Henry III. Tower,
Windsor Castle, October 29.

### THE FLORENCE NIGHTINGALE MEMORIAL.

A meeting was held on Tuesday in the Court Room of St. Thomas's Hospital to consider the establishment of a memorial to Miss Florence Nightingale.

Telegrams were received expressing regret at being detained from the meeting from Lord Sandhurst, Treasurer of St. Bartholomew's Hospital, Lord Cheylesmore, Chairman of Brompton Hospital, and from Lord Goschen, Treasurer of Guy's Hospital. The following were present: Mr. J. G. Wainwright, Treasurer, St. Thomas's Hospital; Mr. Holroyd Chaplin, Chairman, Royal Free Hospital; Miss R. A. Cox-Davies, matron, Royal Free Hospital; Miss E. H. Becher, Queen Alexandra's Imperial Military Nursing Service; Miss Mabel H. Cave, matron, Westminster Hospital; Miss A. McIntosh, matron, St. Bartholomew's Hospital; Miss M. L. Davies, matron, St. Mary's Hospital; Mr. Edmund Boulnois, Chairman, Nightingale Training School; Miss A. Lloyd-Still, matron, Middlesex Hospital; Miss A. Macnab, matron, Brompton Hospital; Miss I. C. Bennett, matron, Metropolitan Hospital; Miss L. V. Haughton, matron, Guy's Hospital; Miss D. Finch, matron, University College Hospital; the Hon. Sydney Holland, Chairman, London Hospital; Mr. Henry T. Butlin, President, Royal College of Surgeons; Miss E. Harte, matron, Royal Hospital, Haslar; Sir J. Wolfe-Barry, K.C.B., Chairman, Westminster Hospital; Miss G. Payne, matron, Hospital for Sick Children; Sir Henry C. Burdett, K.C.B.; Mr. W. Austen Leigh, Chairman, St. Mary's Hospital; Miss A. M. Hall, matron, Seamen's Hospital; Mrs. Florence Lucas, Nurses' Co-operation; Sir James Porter, Medical Director-General, R.N.; Surgeon-General W. L. Gubbins, Director-General A.M.S.; Mr. Perceval A. Nairne, Chairman, Seamen's Hospital; and Mr. A. William West, Chairman, St. George's Hospital.

On the proposal of Mr. J. J. Wainwright it was agreed unanimously that "a fund for providing a memorial to Miss Florence Nightingale, O.M., be established, and that contributions be invited from all parts of the Empire." Mr. Sydney Holland's motion, which was seconded by Mr. H. T. Butlin, "That a statue shall be erected as part of the memorial," was also carried, as was the proposal of Sir James Wolfe-Barry, which was seconded by Sir Henry Burdett, "That this committee should confer with the committee recently appointed by a meeting held at Grosvenor House, which aims at establishing a memorial to Miss Nightingale, with a view to combination and, if possible, a common course of action."

The following agreed to serve on this Committee: Mr. J. G. Wainwright, Treasurer of St. Thomas's Hospital; Sir Thomas Barlow, President of the Royal College of Physicians; Mr. H. T. Butlin, President of the Royal College of Surgeons; Sir J. Wolfe-Barry, K.C.B., Chairman of Westminster Hospital; Mr. A. William West, Chairman of St. George's Hospital and Chairman of the Central Hospitals Council; Sir James Porter, K.C.B., Medical Director-General of the Royal Navy; Surgeon-General W. L. Gubbins, Director-General Army Medical Services; Miss McIntosh, matron, St. Bartholomew's Hospital; Miss L. V. Haughton, matron, Guy's Hospital; Miss Luckes, matron, London Hospital; Miss Hamilton, matron, St. Thomas's Hospital; Miss Becher, matron-in-chief, Queen Alexandra's Imperial Military Nursing Service; Miss E. Harte, head sister, Queen Alexandra's Royal Naval Nursing Service; Miss Sidney Browne, matron-in-chief, Territorial Force Nursing Service.

It should be added that the work of carrying out the proposal for a Florence Nightingale statue, and of considering what further form the memorial should take, was entrusted to a sub-committee, on the suggestion of Miss Hamilton and Miss McIntosh.

A cordial vote of thanks to Mr. Wainwright for calling and presiding at the meeting was passed.

### Elections and Appointments.

The Duke of Norfolk has been re-elected President of the Sheffield Royal Hospital, and Colonel Sir John E. Bingham, Bart., and Sir Frank Mappin, Bart., Vice-Presidents, and Mr. James Henry Doncaster, Hon. Treasurer. The following will constitute the Board for the ensuing year: Mr. Philip K. Wake, Mr. Thomas S. Ellin, Mr. Joseph Binney, Mr. Charles Wardlow, Mr. Harry P. Marsh, J.P., Mr. G. Jackson Smith, Mr. Thomas K. Wilson, Mr. George Howson, J.P.; Mr. G. K. Thorpe, Mr. George A. Parker, Mr. Herbert Bedford, Mr. B. George Wood, Mr. Joseph G. Elliot, Mr. W. Blake Burdekin, Mr. Edward Dixon.

